# Economic evaluation of community acquired pneumonia management strategies: A systematic review of literature

**DOI:** 10.1371/journal.pone.0224170

**Published:** 2019-10-24

**Authors:** Marufa Sultana, Abdur Razzaque Sarker, Nausad Ali, Raisul Akram, Lisa Gold

**Affiliations:** 1 Nutrition and Clinical Services Division, International Centre for Diarrheal Disease Research, Bangladesh (icddr,b), Dhaka, Bangladesh; 2 Deakin Health Economics, School of Health and Social Development, Deakin University, Geelong, Victoria, Australia; 3 Health Economics and Financing Research, Bangladesh Institute of Development Studies (BIDS), Dhaka, Bangladesh; 4 Health Systems and Population Studies Division, International Centre for Diarrheal Disease Research, Bangladesh (icddr,b), Dhaka, Bangladesh; Stony Brook University, UNITED STATES

## Abstract

**Background:**

Community-acquired pneumonia (CAP) is a major cause of mortality and morbidity worldwide. Efficient use of resources is fundamental for best use of money among the available and novel treatment options for the management of pneumonia. The objective of this study was to systematically review the economic analysis of management strategies of pneumonia.

**Methods:**

A systematic search was performed using Academic Search Complete, MEDLINE, EconLit, Global health, MEDLINE complete and Embase databases using specific subject headings or key words in May 2018 without restricting publication year. All search results were recorded and any type of economic evaluation for management of CAP was included for detailed review. The Consolidated Health Economic Evaluation Reporting Standards (CHEERS) checklist was used for quality appraisal.

**Results:**

Nineteen studies met the inclusion criteria; ten studies were trial based, five conducted analysis using model based techniques and the rest of the studies were either based on observational, record review or pre-post intervention studies. Most of the studies conducted cost-effectiveness analysis (n = 15) and compared different combinations of antimicrobials. Most were based on developed countries (n = 17), considered adult age groups (n = 16) and used a provider perspective (n = 14). Nine studies reported dominant alternatives (lower cost with higher benefit). Sensitivity analysis was performed by the majority of studies (n = 15). Fourteen studies were assessed as either being excellent, very good or good quality, with no relationship found between publication year and study quality. Methodological variation, type of microbial used, perspective, costs and outcome measures limit the compatibility among the results of the included studies.

**Conclusion:**

Economic evaluation of interventions for management of CAP to date supports cost-effectiveness of studied interventions. However, evidence relates largely to antimicrobials choice in older populations in developed countries. Parallel economic evaluation of different management strategies of CAP is recommended for both developed and developing countries to support rigorous and robust comparative economic analysis within health care systems.

**PROSPERO registration no**: CRD42018097174

## Introduction

Community-acquired pneumonia (CAP) is a major cause of morbidity and mortality worldwide and is associated with excessive health care consumption and cost [1,2]. Suspected CAP is defined as “acute symptoms and presence of signs of lower respiratory tract infection (LRTI) without other obvious cause, whereas new pulmonary infiltrate on chest radiograph is needed for definite diagnosis” [1]. Despite noticeable improvement in the management of CAP, it still remains as a health burden for both developed and developing countries [3,4]. The 2013 Global Burden of Disease (GBD) study estimated that lower respiratory infection (LRTI) particularly pneumonia remains the second leading contributor of global disability-adjusted life years (DALYs), and accounted for approximately 1,400 age-standardized DALYs per 100,000 population [4]. CAP remains as a leading cause of death for infectious diseases in the United States and Canada [3]. Incidence rates of CAP range from 1.6–11.6 per 1000 in Europe [5]. The incidence rates reported in the Asia-Pacific region have ranged from 0.2–0.9 per 1000 [6], although these may be underestimated [5]. Incidence of CAP by age follows a U-shaped pattern, with the highest incidence amongst under-five years and over 60 years age groups [7]. Depending on clinical presentation, pneumonia is classified as pneumonia, severe pneumonia, or very severe disease according to the 2013 WHO guidelines [8].

Pneumococcal vaccine (PCV) and Haemophilus influenzae type b (Hib) conjugate vaccines have been incorporated in many countries as a preventive strategy for pneumonia [9,10]. Since vaccine efficacy varies and pneumonia is caused by different types of pathogens, cost-effective management strategies are also deemed important to tackle the morbidity and mortality caused by pneumonia. Standard management of pneumonia usually relies on hospital management including oxygen therapy, antibiotics, and careful monitoring [8,11]. A study in the Netherlands found that, among 0.12 million cases of CAP between 2008 and 2011, 63% of cases required hospitalization, with a mean 6.7 days length of hospital stay [7].

Management of pneumonia is associated with significant economic burden, especially for inpatient hospital care [12–15]. The burden is particularly concerning in low-income countries, where a high economic and health burden for governments and households is often combined with limited access to health care facilities and low quality of services provided [4,10]. With limited resources and growing healthcare needs, economic evaluations have become important tools to help policy makers allocating scarce resources efficiently [16–19]. Decision makers require information regarding the effect of interventions or programs in terms of their costs and associated benefits [18]. Therefore, economic evaluation is crucial to identify the treatment options with best value for money through systematic analysis of costs against associated outcomes [19,20]. This evaluation is particularly imperative for low-income setting with high burden of infectious diseases.

Economic evaluation of different interventions of CAP management has been conducted from various perspectives, however, high quality of reporting also needs to be ensured in order to generate evidence with validated results and to inform policy. To our knowledge, there is no systematic review focused on economic evaluation of CAP management strategies. As such, the aim of this study was to conduct a systematic review of economic evaluation of the management of CAP and to provide a comprehensive summary of the economic evidence to date.

## Methods

### Search strategy

A systematic literature search was conducted in electronic databases using indexed subject headings and free text from early-mid May 2018 to identify published articles that provided cost as well as outcome data of pneumonia management. Academic search complete, MEDLINE, EconLit, Global health and MEDLINE complete databases were searched through the EBSCOhost database. Further, a separate search was also conducted through the Embase database. The search strategy used thesaurus and EMTREE as subject headings for EBSCOhost and Embase databases respectively (Supplementary 1). Methods were based on other systematic review studies in the area of economic evaluation and results are reported following PRISMA statement guidelines [21]. The systematic review protocol was registered prior to conduct of the review (PROSPERO Registration Number: CRD42018097174). Available at: http://www.crd.york.ac.uk/PROSPERO/display_record.php?ID=CRD42018097174

### Inclusion and exclusion criterion

All peer reviewed full-text articles available in English that included any type of economic evaluation for management of pneumonia were included in the review. Studies were included in the review when they fulfilled all of the following inclusion criteria:

- Economic evaluation that reported costs and outcomes of at least two alternatives
- Any type of economic evaluation (i.e., cost-minimization analysis [CMA], cost-effectiveness analysis [CEA], cost-utility analysis [CUA], cost-benefit analysis [CBA])
- Clinical management of CAP
- Published in any year
- Based on studies done alongside randomized clinical trials (RCTs) or observational studies, or modelling studies. Full text journal articles
- Published in English or other languages if English version available
- Studies were excluded from the review when they met any of the following exclusion criteria:
- Partial economic evaluation, i.e. with no comparison group, or only cost analysis of management of pneumonia
- Only clinical outcome/effectiveness or benefit using non-monetary terms (no costs)
- Economic evaluation other than treatment of pneumonia (for example, preventive intervention for pneumonia such as vaccination)
- Books, reports, reviews, letters, editorials, perspectives, abstracts, and methodological articles

### Selection process

Search results were initially screened against inclusion/exclusion criteria on the basis of the title, abstract and publication details. Studies that were not relevant to pneumonia management or cost-effectiveness; and conference, editorial, commentary and review studies were excluded at this stage. Different types of economic evaluation studies that focused on vaccination for preventing pneumonia were also excluded. Screening was undertaken by three researchers independently (MS, RA, NA) and a study was excluded on title and abstract only if all reviewers agreed. Any disagreements were resolved by evaluating the full-text of the paper (MS, ARS, RA, NA). Full text of potential studies for inclusion were obtained and assessed against inclusion criteria.

### Data extraction and synthesis

For each of the included studies, data were independently extracted by two reviewers (MS and LG) using a formatted spreadsheet in Microsoft Excel. Data extraction followed guidance of ‘Cochrane Handbook’ chapter on economic evidence [22]. As such, following data were extracted regarding the characteristics of each economic study: study year and study period, detail of intervention and comparators, study design and source(s) of resource use, study population, study location/country and setting, unit costs of resources used with currency and price year, type of economic evaluation, time horizon of both costs and effects, analytical viewpoint, effectiveness data, sensitivity analysis, decision-making statements, and limitations. For results, resources used and unit costs estimates associated with interventions and comparators were extracted separately. Outcome data such as LOS in days, follow-up periods, patients and/caregivers’ time were extracted as natural units [22]. Meta-analysis was not conducted in this review. Resource use and outcome data were not pooled since outcomes were different across RCTs and other studies. In addition, a range of methodological variations, country specific results, differences between health systems and study perspective restricted quantitative comparability across studies. Therefore, a qualitative narrative i.e. comprehensive synthesis that focused on critical appraisal and discussion of the major findings of was of included studies was employed. All the costs identified within included studies were converted to 2018 US dollars (US$) to increase compatibility of the results [22]. To accomplish this, cost data were converted to international dollar units (US dollar value) using EPPI-CCEMG Cost calculator (https://eppi.ioe.ac.uk/costconversion/). As per earlier study, cost data were set to one year prior to publication year to convert if year was not stated [17]. The study characteristics are summarized in tables and figures.

### Quality (risk of bias) assessment

The quality of included studies was evaluated against 24 key criteria specified in the Consolidated Health Economic Evaluation Reporting Standards (CHEERS) checklist [23]. This recently-published checklist, consisting of 24-item, was supported by the International Society for Pharmacoeconomics and Outcomes Research (ISPOR), BMJ and other publications [23]. This checklist includes six major areas: title and abstract (two items); (ii) introduction (one item); (iii) methods (14 items); (iv) results (four items); (v) discussion (one item); and (vi) other, which is related to funding and conflict of interest (two items) those need to be reported in economic evaluation studies [23].

A score was given for each applicable item and a total score (out of 24) was obtained for each study. Studies that fully met each of the items of the checklist were scored as ‘1’, those deemed to have partially met each of the criteria were scored as ‘0.5’ and scored ‘0’ if studies did not meet the criterion at all [19]. Therefore, total score was obtained ranging from 0 to 24 points. Two reviewers (MS and LG) discussed the ratings before final calculation of the score. Based on earlier studies, studies were considered as “excellent quality” of reporting with a score higher than 85%, 70–84% as “very good quality”, 55–69% as “good quality” while studies with lower than 55% were considered as “poor quality” [19,24].

## Results

### Search results

**Fig 1:**
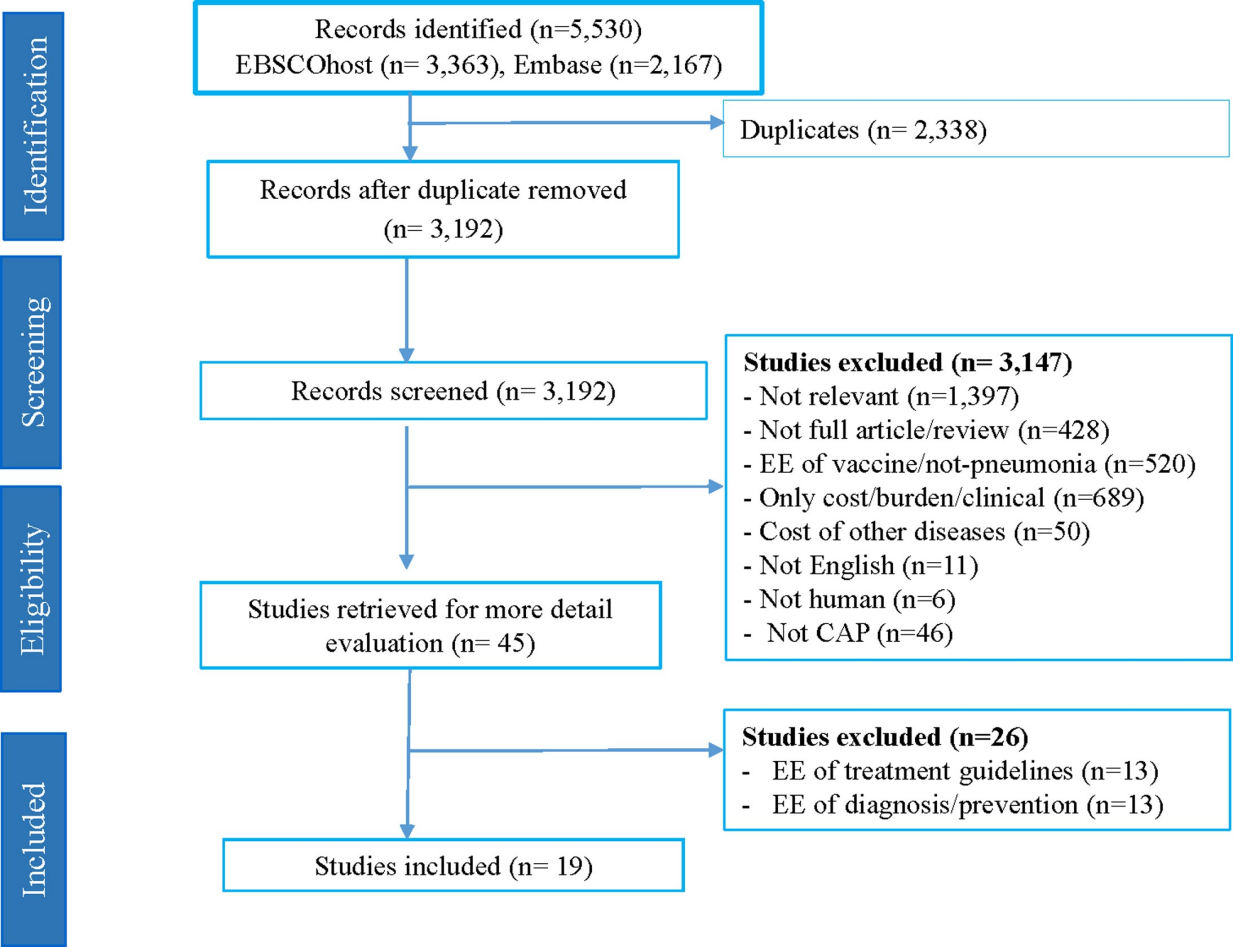
Flow chart of literature search (PRISMA) for economic evaluation studies.

Fig 1 describes the search results and selection process. The initial searches through EBSCOHost and Embase databases yielded 3,363 and 2,167 titles and abstracts respectively. Duplicates were removed from the identified articles. Publications clearly not meeting inclusion criteria were excluded on screening of title and abstracts (n = 3,147). A further 26 papers were excluded after reviewing the full text. Finally, nineteen peer–reviewed eligible articles were included for detailed review.

### Characteristics of the selected studies

The characteristics of the included articles are summarized in Table 1. Most studies performed cost-effectiveness analysis (n = 15) and were conducted in developed countries (n = 17) i.e. United States (n = 7), Germany (n = 4) or other European countries (n = 6). Only two studies were conducted in a low and middle income country context (Malawi and Malaysia) [25,26]. Studies were mainly conducted from a hospital/government, health system, or third party perspective (n = 14), while two studies reported a broader societal perspective [27,28]. Ten studies were based on randomized controlled trials, five studies were model based and the remaining 4 studies were either observational or pre-post intervention analysis. The sample size of included studies ranged from 28 to 2,283 (median 337). Most studies considered adults (n = 15; mean age ranged from 48–72 years); two studies were based on under-five children [25,26] and one study considered all age groups [29]. Publication years ranged from 1998 to 2017, with 84% (n = 16) of the studies published before the publication of the CHEERs checklist in 2013.

**Table 1:** Summary of study characteristics.

Study Characteristics	No of studies	Study design		
***Type of Economic evaluation***		**RCT**	**Model**	**Pre-post/others**
CMA	3	2	0	1
CEA	15	8	4	3
CUA	1	0	1	0
***Perspective ***				
Societal	2	2	0	0
Hospital/government	9	5	1	3
Health system/third party payer	5	1	3	1
Not specified	3	2	0	1
***Population ***				
All age groups	1	0	1	0
<5 years	2	1	1	0
≥5 years	15	9	3	3
Not specified	1	0	0	0
***Main treatment method evaluated***				
Staff education with antibiotics	1	0	0	1
Single or combined doses of oral/IV antibiotics	17	9	5	3
BCPAP with antibiotics	1	1	0	0
***Comparison group ***				
Existing practice	2	1	1	0
Another specific treatment group	12	9	0	3
No intervention	5	0	5	0
***Year of publication ***				
1998–2012	16	9	3	4
2013–2018	3	1	2	0
***Country according to income level ***				
High income countries	17	9	4	4
Low and middle income countries	2	1	1	0

Most studies compared the efficacy of different combination of antibiotics, ranging from 1st-3rd generation antibiotics (penicillin, gentamycin, cephalosporine, fluroquinolone, macrolides, carbapenem, B-lactum etc.), with associated costs (n = 12). Two studies evaluated other interventions along with recommended antibiotics; one described usual management of pneumonia with an intervention that combined staff education, visual reminder and manual [30] and the other compared usual care with the bubble continuous positive airway pressure (bCPAP) alongside other supportive care [26] (Table 2).

**Table 2:** Summary of the economic evaluation studies on management of pneumonia.

Author (Year)	Analysis	Perspective	Country	Population, Sample size (SS)	Study design	Intervention &/Comparators	Main cost measurement	Cost/outcome measure	Currency, year	Sensitivity analysis	Main results (2018 USD)	CHEERS score and quality
van Barlingen et al. (1998) [29]	CEA	Health system	Germany	All age with CAP, SS: NS	DA model (modified delphi technique);	3 first line antimicrobials: macrolides, fluoroquinolones, and cephalosporins	Outpatient medication, consultation, diagnostic procedure and tests, number of hospital days	cost per successfully treated patient	Euro, 1996 (German tariff)	Several parameters used from delphi panel and literature;moderate CAP is very sensitive for first line success rate due to high hospitalization costs for treatment failure	As per cost per measure **Mild CAP:** fluoroquinolones<macrolides<cephalosporins: US$404<US$432<US$506 **Moderate CAP:** cephalosporins<fluoroquinolones<fluoroquinolones: US$1,647<US$1,713< US$2,189.	14 (good)
Dietrich et al. (1998) [31]	CEA	Hospital & insurer	Germany	>18 years with CAP including exclusion criterion, SS: 604	Observational, multisite	Third generation cephalosporin (ceftriaxone) compared with a second-generation cephalosporin (cefotiam/cefuroxime)	Antimicrobials preparation, administration, dispensing and drug costs, LoS, side effects, diagnostic tests and other drug costs	cost per successfully treated patient	Deutsche Mark (DM) and USD, 1998	Threshold calculated; ceftriaxone remained cost-effective with assumed changes of personnel, laboratory and other costs.	Significant difference in success rate between two antibiotic groups (81.4% vs 91%, p < .0001). Cost lower with third generation (ceftriaxone) due to lower preparation and administration costs that led lower per patient cost (US$154 Vs US$ 386).	10 (poor)
Caldwell et al. (1999) [34]	CEA	Hospital	USA	>18 years with severe pneumonia, SS: 27	RCT	Ciprofloxacin compared to imipenem	Costs of antimicrobials, hospital perdiam, unit costs from hospital rates	cost per patient cured	USD, year not specified	Not presented	Clinical resolution was 77% and 50% for Ciprofloxacin and imipenem group respectively. Cost per case cured was 2.6 times higher for imipenem (US$ 42,520Vs US$ 111,433 for Ciprofloxacin Vs imipenem).	8.5 (poor)
Rittenhouse et al. (2000) [43]	CEA	Not specified	USA	>18 years with CAP including exclusion criterion, SS: 211	RCT	Oral levofloxacin with oral cefuroxime Axetil	Drug costs, OP-ED-hospital care including tests. Unit costs from Medicare fee schedule, or estimated.	Clinical improvement	USD, 1997	Excluded one outlier for sensitivity analysis (patient receive Rx <48 hours); Included costs not related to pneumonia	Drug cost was lower for levofloxacin (US$136 vs US$ 264) both for base case and sensitivity analysis. Overall difference not significant (US$1,061 vs US$ 1309).	8 (poor)
Dresser et al. (2001) [32]	CEA	Hospital	USA	>18 years with CAP including exclusion criterion, SS: 201	RCT	IV fluoroquinolone (gatifloxacin) with IV cephalosporin (ceftriaxone) with or without IV erythromycin to oral clarithromycin	Antimicrobials preparation and administration, dispensing and drug costs; costs of side-effects; hospital per diem costs. CAP-specific hospital per diem estimates	Mean cost per expected success	USD, not specified	Plausible ranges tested in sensitivity analysis; economic decision was not altered after sensitivity analysis employed	No significant difference found in treatment success rate between groups (97% vs 91%). Cost per outcome lower for gatifloxacin than ceftriaxone thus cost-effective (US$7,450 Vs US$ 8,527).	13.5 (good)
Paladino et al. (2002) [38]	CEA	Hospital	USA	>18 years with CAP including exclusion criterion, SS: 266	RCT	IV/oral azithromycin Vs cefuroxime with/without erythromycin	Antimicrobials preparation, administration, dispensing and drug costs; diagnostic tests, hospital stay costs, weighted DRG perdiam	Cost effectiveness ratio per expected cure	USD, year not specified	Several key parameters used for sensitivity analysis; Costs constantly lower for azithromycin thus economic decision unchanged.	No significant difference in treatment success (78% vs 75%). Overall cost was lower for azithromycin (high purchase price but lowers LoS, duration of treatment and admin cost) thus cost-effective.	13.5 (good)
Drummond et al. (2003) [36]	CEA	Health system	France and Germany	>18 years with CAP including exclusion criterion, SS: 622	RCT	IV fluoroquinolone (moxifloxacin) Vs IV/oral co-amoxiclav with macrolide	Drug costs, hospital stay, out-of-hospital care (tests, X-rays & drugs).	Cost per case cured, probability of cost saving	Euro, 2000–2001	One-way sensitivity analysis using several parameters; -acceptability thresholds showed moxifloxacin is cost-effective or cost-saving	ITT analysis showed cure rate 93% vs 85% for intervention and control groups. Cure rate was8.3% higher in intervention (moxifloxacin group). Treatment with moxifloxacin was less costly than the amoxiclav regimen by US$765 and US$ 1,095 in Germany and France but difference non-significant.	16 (very good)
Frei et al. (2005) [40]	CEA	Hospital	USA	>18 years with severe pneumonia, SS: 393	case record review	4 groups compared by first line Antimicrobials: levofloxacin, ceftriaxone, ceftriaxone+macrolids, ceftriaxone+ levofloxacin	Charge for hospital stay (drugs, diagnostic tests, emergency department)	cost-effectiveness ratio	USD, 2005	Probabilistic sensitivity analysis on cost-effectiveness ratios; levofloxacin was most cost-effective	Per patient cost US$6,411. Cost varied among drug groups but no significant difference.	12 (good)
Hasali et al. (2005) [25]	CEA	Not specified	Malaysia	Children 2–59 months with moderate CAP, SS: 40	RCT	IV ampicillin Vs IV/ampicillin+IV gentamycin	Drug costs, drug administration costs, lab costs, hospital stay, staff costs	Cost per patient	Malaysian Ringgit, 2000	Not presented	Per patient cost lower for ampicillin than combined (US$159 Vs 247). LoS, staff costs, drug costs, admin and hospital costs lower in intervention	10.5 (poor)
Samsa et al. (2005) [27]	CMA	Societal	USA	>18 years with CAP including exclusion criterion, SS: 163	RCT	IV/ oral azithromycin Vs IV/oral levofloxacin	Drug costs, hospital stay (by ward), home care, out-of-hospital HC use, productivity loss.	Cost per patient	USD, 2002	100 bootstrap samples with replication; 98 exceeded per patient cost of intervention	Cost per patient higher among levofloxacin group (Azithtromycin vs levofloxacin: US$12,630 vs 16,008). Difference was due to lower LoS.	12 (good)
Barlow et al. (2007) [30]	CEA	Hospital	Scotland	>16 years with CAP including exclusion criterion, SS: 503	Pre-post with intervention and control	CAP management pathway (staff education sessions, visual reminders, manuals) compared to usual care	1st Antimicrobials dose; intervention costed mainly by staff time)	cost per death prevented	GBP, 2002	Intervention cost and scale up cost included for sensitivity analysis	Patients receiving appropriate antibiotics within 4 hours of admission increased by 17% (adjusted). Cost per additional patient was US$ 261.	14.5 (good)
Bhavnani et al. (2008) [33]	CEA	Hospital	Multicountry (mainly North America)	>18 years with CAP without immunocompromised, SS: 341	RCT	oral gemifloxacin Vs intravenous ceftriaxone followed by oral cefuroxime with or without a macrolide	Antimicrobials preparation, administration, dispensing and drug costs; diagnostic tests, hospital stay costs, cost of side effects	Median cost per expected success	USD, 2004	Not presented	No significant difference among clinical success (77% vs 79%. Cost lower for gemofloxacin in all 1^st^, 2^nd^ and 3^rd^ level treatment. Cost per expected success lower for gemofloxacin thus cost effective (non-significant)	11.5 (good)
Lloyd et al. (2008) [37]	CMA	Hospital and insurer	Multicountry (17 countries in Europe, Latin America and South Africa)	>18 years with CAP required hospitalization including exclusion criterion, SS: 733	RCT	IV/oral moxifloxacin compared to IV/oral levofloxacin plus ceftriaxone	Drug costs, hospital stay, in-hospital tests & procedures, readmission.	per-patient expenditure	Euro, Unit costs from German 2006 CPI value	Presented including difference in costs and outcomes to generate ICER but no significant difference between two treatment groups; ICER of US$ 15,687 per additional patient cured for moxifloxacin.	80% vs 84% resolution for intervention and control respectively. LoS similar. Cost US$3,369 vs US$4,029, significant difference mainly due to lower drug costs. Cost was US$ 4,128 vs US$ 4,106 from insurer perspective, difference was not significant.	16 (very good)
Martin et al. (2008) [42]	CEA	Third party payer	Belgium	Not specified, patients with CAP, SS: NS	DA model	Comparison of 4 1st-line Rxs: fluoroquinolone (moxifloxacin), B-lactam (co-amoxiclav, cefuroxime), macrolide (clarithromycin)	Drug costs, GP/specialist/ED costs; X-rays/tests, hospital stay.	CER and ICER with different first to second line outcome measures	Euro, Unit costs from Belgium 2006 value	Both probabilistic and deterministic sensitivity analysis were carried out with multiple parameters. Moxifloxacin found as dominant	Costs per episode amounted to US$222 with moxifloxacin, US$ 342 with co-amoxiclav, US$ 325 with cefuroxime, US$ 297with clarithromycin. The moxifloxacin/co-amoxiclav strategy offered lower rates of first-line clinical failures, rehospitalisations, and mortality than all comparators.	14 (good)
Lee et al. (2009) [44]	CMA	Not specified	Hong Kong	>18 years with CAP including exclusion criterion,SS: 333	case record review	3 initial Rx: amoxycillin-clavulanate (AC); AC+macrolide (ACM); quinolone (Q)	Drug costs, consultation costs.	Mean cost per patient	Hong Kong Dollar, unit costs from tariffs (2003, 2007)	Not presented	Mortality lower for ACM than AC/Q. Overall cost lower in Q (US$11,118) vs AC (US$14,539) or ACM (US$16,250) but non-significant difference	11 (poor)
Edwards et al. (2012) [39]	CUA	Health system	UK	Adults with severe pneumonia in critical care (mean age 68 years), SS: NS	Markov model	carbapenem (meropenem) with penicillin (piperacillin/ tazabactam)	Drug costs, drug prep, dispensing & admin costs; hospital stay by type of ward & level of support; OP visits.	ICER	GBP, Unit costs from UK tariffs 2008	Probabilistic sensitivity analysis was done using different parameters; meropenem dominates consistently	4.77 vs 4.66 QALYs for intervention and control. Cost per patient estimated to US$32,345 vs 33,858; so intervention cost-effective. PSA supports meropenem (cost-saving in >90%)	20.5 (excellent)
Grau et al. (2014) [41]	CEA	Health system	Spain	>65 years with severe pneumonia, SS: 1000 hypothetical cohort	DA model	Carbapenem (ertapenem) with 3rd generation cephalosporin (ceftriaxone)	Drug costs, hospital stay.	Difference in proportion of cost and treatment success	Euro, Unit costs from Spanish 2006 CPI value	One-way and probabilistic sensitivity analysis was done; length of stay was found as the key parameter	Clinical success 71% vs 65% for ertapenem Vs ceftriaxone. Drug costs US$ 720 vs US$ 411 but overall cost US$ 7,634 vs US$ 7993; ertapenem dominant in 59% of PSA.	15 (very good)
Kortz et al. (2017) [26]	CEA	Govt. hospital	Malawi	Children aged 1 month–5 years with severe pneumonia, SS: NS	DA model	Bubble continuous positive airway pressure (bCPAP) plus supportive care compared to usual care	bCPAP costs (including training, pulse oximetry & NP suction). Hospital per diem cost.	averted DALYs, ICER	USD, year not specified (unit cost from malawi)	A series of one-way sensitivity analysis	Cost per episode was US$155 vs U$ $90 for BCPAP Vs usual care. DALYs calculated as 2.4 vs 7.4. Cost was -US$13/DALY averted so BCPAP is cost-effective.	18 (very good)
van Werkhoven et al. (2017) [28]	CMA, CEA	Societal	Netherlands	>18 years with CAP including exclusion criterion, SS: 2,283	RCT	Beta-lactam/ macrolide or fluoroquinolone compared to beta-lactam monotherapy	Drug costs, hospital stay & tests; out-of-hospital HC use, days out of role, patient costs (travel), carer days out of role.	Cost per patient, ICER for cost per death prevented	Euro, 2012 (Unit costs from Dutch sources)	Not presented	Non-significant difference outcomes in trial. 90-day costs were US$6,124 vs US$6,264 vs US$ 5,707 for comparators, but difference not significant	15.5 (very good)

### Reported cost of the studies

Reported costs were mainly based on the chosen perspective and the nature of the study (trial or model based). All studies reported direct costs of the treatment, with most commonly reported costs related to antimicrobial drug costs (including preparation, dispensing and administrative costs). In general, direct costs also included hospital per diem/stay costs, physician costs, costs of other drugs, laboratory tests, and staff training costs. Cost of side effects was considered by three studies [31–33]. The two studies reporting a societal-perspective analysis estimated loss of productivity (days out of role), home care and out-of-hospital resource use costs (tests, X-rays and drugs) [27,28]. Only van Werkhoven et al. included non-health care costs (travel cost) in cost analysis [28]. Detailed methodology of cost analysis was not described by two studies [30,34]. Unit costs were mainly sourced from local tariffs, medical records, experts’ opinion and published literature. Multi-country trials presented cost data in a single currency [35–37]. Currency was reported in all studies, however, reporting year was not specified by four studies [26,32,34,38]. Due to the short time periods involved, most studies did not apply discounting for cost analysis (n = 17), five studies provided this justification [32,39–42]. One study applied a 3% discount rate for cost and outcome without specifying the time horizon considered, another study discounted productivity loss at a rate of 3% if it was longer than a year but no discounting for cost was applied [26,28].

It is important that an evaluation is conducted over a time horizon that includes all relevant costs and consequences from the chosen perspective of the study [22,23]. More than half of studies (n = 10) specified the time horizon/duration of the treatment, with follow-up that ranged from 21 days to lifetime. However, eight studies (42%) specified their duration of the study period only. Sensitivity analysis was performed by 78% of the studies (n = 15) and length of hospital stay (LoS) was commonly reported as the most cost-sensitive parameter.

### Summary results of effectiveness and economic evaluation

The economic evaluation studies selected for this review were conducted in different countries and the evaluated management strategies included different combinations of antibiotics. In addition, study design, type of intervention(s) and description of the usual care also differed widely. In this narrative review, we found that Intention to treat (ITT) approach was applied by all studies, with 11 studies imputed missing data for analysis. Outcomes were mainly reported as clinical success (defined as cured or condition improved), deaths prevented, mortality averted, Quality Adjusted Life Years (QALYs) gained and DALYs averted. None of the included studies conducted cost-benefit analysis.

Trial or pre-post outcome based studies (n = 14) expressed economic evaluation outcomes as cost per patient (n = 5, 26%) [24,26,27,36,42], cost per case cured (n = 3, 16%) [31,34,36], cost per expected success (n = 4, 22%) [32,33], cost per clinical improvement (n = 1, 6%) [43], and cost per death prevented (n = 1, 6%) [30]. Comparators included different combinations of antibiotics, usual care, and intervention based on staff education. Of the studies assessing cost per patient (n = 5), three studies performed CMA [27,37,44], one conducted CEA [25] and one study combined both CMA and CEA [28]. For CMA studies, two studies reported lower costs with equivalent outcomes particularly for drugs with lower purchase costs or reduced hospital stay [27,37]. van Werkhoven et al. employed both CEA and CMA for the economic analysis but found no significant differences in costs or outcomes among treatment modalities [28] (Table 2). Among the other CEA studies (n = 11), two studies found dominant results [31,32], where one intervention was both more effective and less costly [31]. Several other studies found no significant difference in outcomes, despite this, cost differences found to be significant in some studies [32] but not for others [38,40] (Table 2). (Table 2). Even though no significant differences was observed between treatment groups, nine studies showed low-cost alternatives among the included studies [28,30,33,34,36,38,40,43,44].

Model-based economic evaluations (n = 5) mostly applied a decision analytic model (n = 4) [26,29,41,42]; one used a Markov model [39]. The incremental cost-effectiveness ratio(s) (ICERs) were calculated by three studies, of which each study found one intervention strategy to be dominant due to higher effectiveness with lower cost [39,41,42]. Kortz et al. determined that bCPAP was cost-effective against current benchmarks, at an ICER of US$13 per DALY averted compared to usual care [26]. The study conducted by van Barlingen et al. modelled cost-effectiveness of three different antibiotics for management of mild and moderate CAP and highlighted the importance of first-line clinical effectiveness as the key cost driver for moderate CAP [29]. Among all included studies, one study conducted cost utility analysis and presented QALYs as a utility measurement (based on utility scores from the EuroQoL EQ-5D) [39].

Comparing model-based and trial-based studies, noticeable differences were identified in reporting results. In particular, significant differences in costs were reported by the majority of the selected model-based analysis (three out of five) compared to trial based interventions (four out of ten). Although most of the trial based interventions reported numerically lower costs with one strategy, these differences were often not statistically significant. This was not simply related to the sample size of trials: among the eight trial-based studies with sample size less than 300, three studies reported significant cost differences between intervention and comparator(s) [25,27,32].

All except one of the model based studies compared different antimicrobials. All model based studies conducted sensitivity analysis while four trial based studies did not specify sensitivity analysis. In most cases, neither type of studies specified time horizon or used discounting due to the short time treatment and follow-up periods involved. A societal perspective was considered only by trial based studies (n = 2), where one showed significant results [27,28].

### Quality of reporting

**Fig 2:**
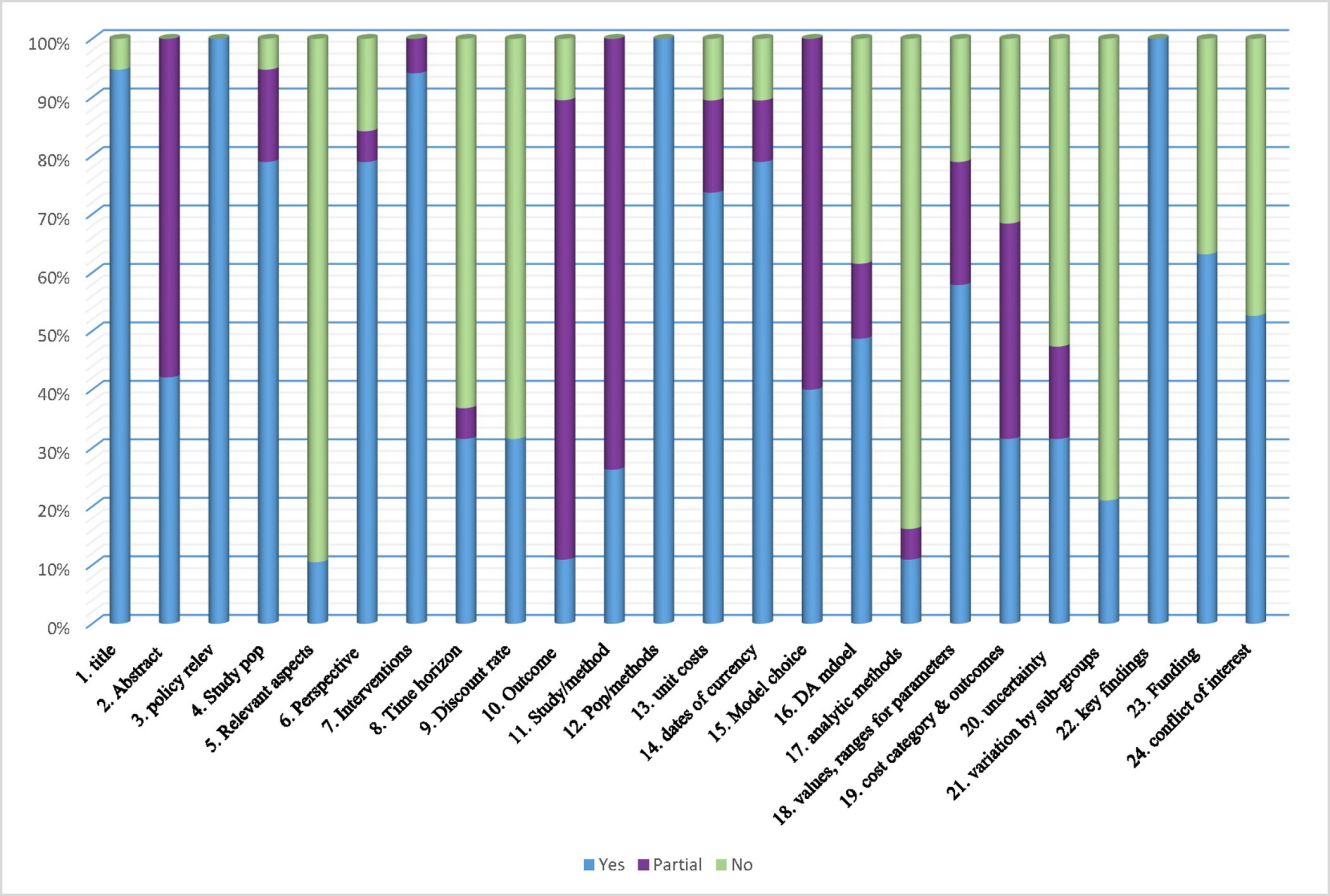
Percentage of the studies appropriately /partially address each item of CHEERS checklist.

The reporting quality of the 19 studies is summarized in Fig 2 and Table 3. Almost all studies appropriately described title, methods, interventions, key findings and policy relevance and partially fulfilled the detailed abstract section. Fig 2 represents the number of studies that fulfilled or partially addressed each recommendation of the CHEERS checklist. The least appropriately addressed items by studies included relevant aspects of system(s), analytic methods (e.g. no detailed methods of handling missing data, pooling or extrapolation, model validation and uncertainty), and description of sub-group analysis. Most frequent partially addressed items included abstracts (did not specify perspective in abstract), outcome measures (justification not provided), methodology (justification for effectiveness data source not stated), and choice of model (no justification). Just over half of included studies described item 23 (study funding and role of funder) and 24 (description of conflict of interest), which might not be stated due to variation in journal requirements.

**Table 3:** CHEERS checklist per item of all included studies.

Checklist		van Barlingen et al.	Barlow et al.	Bhavnani et al.	Caldwell et al.	Dietrich et al.	Dresser et al.	Drummond et al.	Edwards et al.	Frey et al.	Grau et al.	Hasali et al.	Kortz et al.	Lee et al.	Lloyd et al.	Martin et al.	Paladino et al.	Rittenhouse et al.	Samsa et al.	van Werkhoven et al.	No. of Yes	No. of No	Partial
**Title/Abstract/Background**																							
1	Identified as EE in title	Y	Y	Y	Y	Y	Y	Y	Y	Y	N	Y	Y	Y	Y	Y	Y	Y	Y	Y	18	1	0
2	Abstract incls aim perspective, methods, results, conclusions	P	Y	P	P	Y	P	Y	P	P	P	P	P	Y	Y	Y	Y	P	P	Y	8	0	11
3	Study Q & policy relev stated	Y	Y	Y	Y	Y	Y	Y	Y	Y	Y	Y	Y	Y	Y	Y	Y	Y	Y	Y	19	0	0
**Methods**	** **																						
4	Study population & sub-groups described, with why sub-gps chosen	Y	Y	Y	Y	Y	Y	Y	Y	Y	Y	Y	Y	Y	Y	N	Y	P	P	P	15	1	3
5	Relevant aspects of system(s) stated	Y	Y	N	N	N	N	N	N	N	N	N	N	N	N	N	N	N	N	N	2	17	0
6	Perspective stated & related to costs included	Y	Y	Y	N	Y	Y	Y	Y	Y	Y	N	Y	P	Y	Y	Y	N	Y	Y	15	3	1
7	Interventions described & choice explained		P	Y	Y	Y	Y	Y	Y	Y	Y	Y	Y	Y		Y	Y	Y	Y	Y	16	0	1
8	Time horizon stated & justified	N	Y	N	N	N	N	Y	Y	N	Y	N	Y	N	P	N	N	N	N	Y	6	12	1
9	Discount rate stated & justified	N	N	N	N	N	Y	N	Y	Y	Y	N	Y	N	N	N	N	N	N	Y	6	13	0
10	Outcome measures stated & relevance described	Y	P	P	P	P	P	P	Y	N	P	P	P	P	P	P	P	N	P	P	2	2	15
11a (Single study)	Study described & why this sufficient for effectiveness data	N	P	P	P	P	P	P		P		P		P	P		P	P	P	P	0	1	14
11b (Model)	Methods for identification & synthesis described	Y							Y		Y		Y			Y					5	0	0
12	Pop/methods for preference elicitation (if applic)								Y				Y								2	0	0
13a (Single study)	Methods for resource use & unit costs & adjustments	Y	N	Y	Y	Y	Y	Y		Y		P		P	Y		Y	P	Y	Y	11	2	3
13b (Model)	Methods/data sources for resource use & unit costs	Y							Y		Y		Y			Y					5	0	0
14	report dates of currency & methods for adjusting if approp	Y	Y	Y	Y	Y	Y	Y	Y	Y	Y	N	Y	P	Y	Y	N	P	Y	Y	15	2	2
15	Model choice: describe & justify DA model choice, show model fig	Y							Y		P		P			P					2	0	3
16	Detail structural/other assumptions of DA model	N							Y		N		N			P					1	3	1
17	Describe analytic methods incl skewed/missing data, extrapolation, pooling, model validation, uncertainty	N	N	N	N	N	N	Y	N	N	N	N	N	N	N	N	P	N	N	Y	2	16	1
**Results**																							
18	Report values, ranges for all params & (if applic) prob dn (w reasons for dn)	N	Y	Y	N	N	Y	Y	Y	Y	Y	Y	Y	P	P	P	Y	N	Y	P	11	4	4
19	Report each main cost category & outcomes for each option & mean diff and ICER (if applic)	P	Y	Y	N	N	Y	Y	Y	N	P	P	P	N	Y	N	P	P	P	N	6	6	7
20a (Single study)	Describe impact of sampling uncertainty for dC, dE & ICER and impact of assumptions		N	N	N	N	N	Y		N		N		N	Y		P	N	P	P	2	9	3
20b (Model)	Describe impact on results of uncerty in params & assumptions	Y							Y		N		Y			Y					4	1	0
21	(If applic) Describe variation by sub-gps or other variability in results not reducible by more info	Y	N	N	N	N	N	N	N	Y	N	N	N	Y	Y	N	N	N	N	N	4	15	0
**Discussion**																							
22	Summarise key findings & say how support concln. Give ltns, general'y & how fits literature	Y	Y	Y	Y	Y	Y	Y	Y	Y	Y	Y	Y	Y	Y	Y	Y	Y	Y	Y	19	0	0
**Other**																							
23	Study funding, role of funder, other non-$ support noted	N	Y	N	N	N	N	Y	Y	N	Y	Y	Y	N	Y	Y	Y	Y	Y	Y	12	7	0
24	Describe CoI as per jnl policy	N	Y	N	N	N	N	N	Y	N	Y	Y	Y	Y	Y	Y	Y	N	N	Y	10	9	0
	**Score**	13.5	14.5	11.5	8.5	10.0	13.5	16.0	20.5	12.0	15.0	10.5	18.0	11.0	16.0	14.0	13.5	8.0	12.0	15.5			
	**Percentage**	61.4	69.0	54.8	40.5	47.6	61.4	76.2	85.4	57.1	65.2	50.0	75.0	52.4	76.2	60.9	64.3	38.1	57.1	73.8			

***Note*:** *A score for 1 symbolized as ‘Y’ with appropriate reporting of the checklist*, *a score of 0*.*5 symbolized as ‘P’ that partially met the criterion*, *a score of 0 symbolized as ‘N’ if not reported at all*.

Considering the scoring applied for this review, one study was found to be excellent (≥ 85% score), four studies were rated as very good reporting quality (scoring 70–84%), with the remaining studies rated as good (n = 9) or poor (n = 5) quality reporting.

Although the quality of reporting varied within any category of study, model-based analyses rated more highly (average score 68%) than studies based on trials (average score 52%) or other study types (average score 49%). There was also some pattern of quality of reporting by reported cost-effectiveness results: the two studies that reported both higher costs and improved outcomes [26,30] had an average rating of 68%, compared to studies that reported dominant results (average score 56%, score range 35–85%) or non-significant results (average score 52%, score range 33–67%).

**Fig 3:**
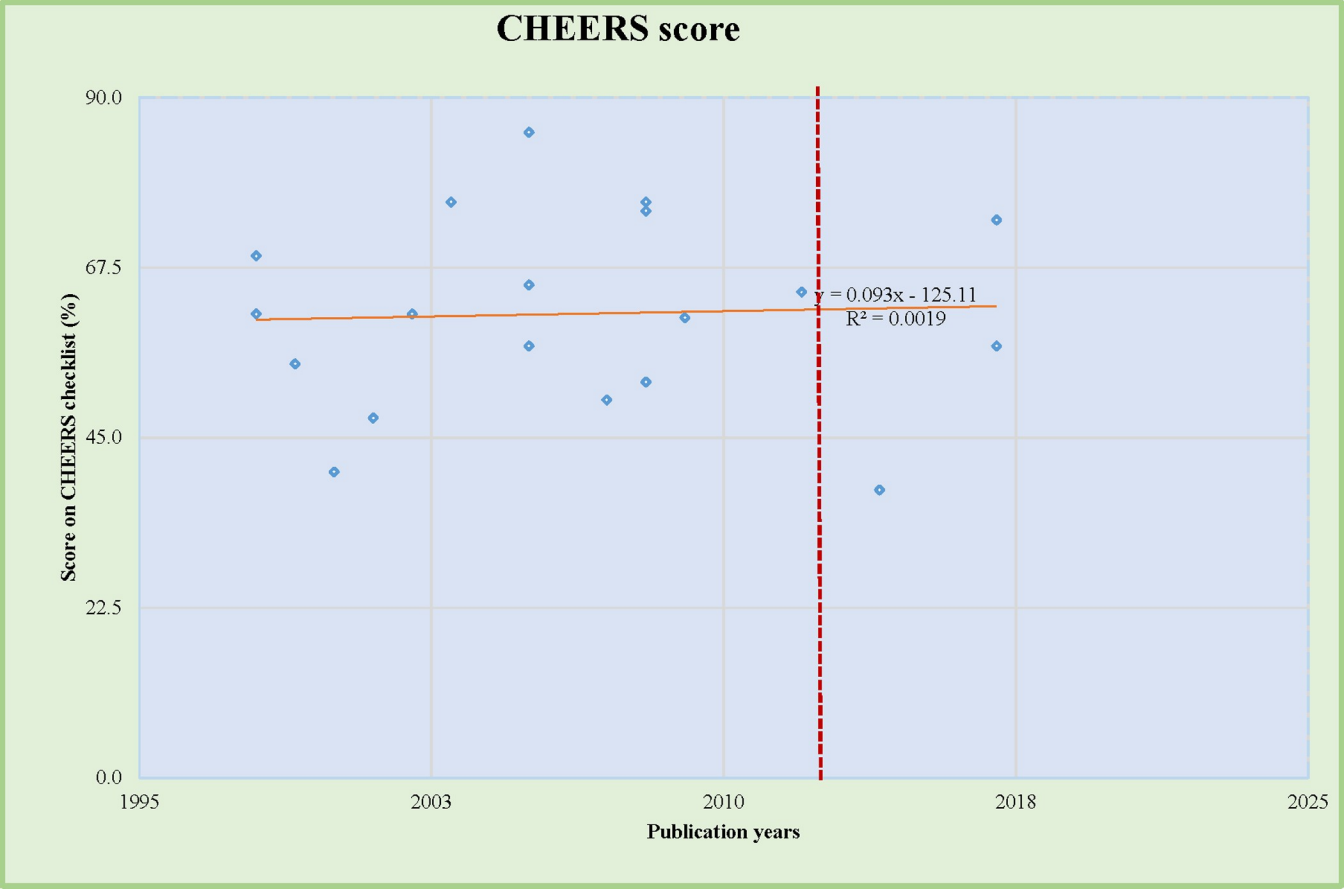
CHEERS score and the trend based on publication years.

Fig 3 shows the linear association between CHEERS score of each study and the publication date to determine any association between quality of reporting and publication years after modification of the checklist in 2013. The linear trend line shows no specific pattern related to study year and reporting quality (R^2^ = 0.001).

## Discussion

The intention of this review was to provide an overview of the current evidence regarding the economic evaluation of different management strategies for pneumonia. This review included 19 studies, mainly conducted in developed countries and focused on adult patients. Only two studies were conducted in LMICs [25,26]. The objective for most studies was evaluation of cost-effectiveness of different novel antibiotics from a provider or government perspective; only two studies adopted a broader societal perspective [27,28]. Most of the identified studies found that the newer antibiotics and/or interventions used for the management of pneumonia-exemplified value for money either in terms of cost-effectiveness against locally-relevant thresholds or by being cost-saving. Despite similarities in reported outcomes across studies, differences exist in assumptions, costs, currency and types of drugs used. These methodological challenges for comparison across studies are common in reviews of economic evidence [17]. This variation acts as barrier for other researchers as there is no clear consensus on what for components to include in cost and cost-effectiveness analysis. Comparing results across studies of different methodological approaches can highlight the importance of certain evaluation choices. For example, studies those included treatment costs for the side effects of drugs [31,32] reported a different cost profile than those that excluded these cost components. Due largely to the short time horizon of studies, most of the studies reviewed here did not consider the costs and effects of long-term follow up of drug treatment, which might cause over- or under-estimation of the drug costs compared to a longer time horizon.

In this review, most studies adopted a health system or hospital perspective for evaluation, while only two studies presented a broader societal perspective [27,28]. This is somewhat different to other reviews of economic evaluations, which have found societal perspective to be more common in other clinical areas, such as mental health [16].

Few studies described costs in detail by incorporating drug preparation costs, administration costs, costs of side effects, diagnostic tests costs, hospital stay and other relevant costs [25,32,33,36,38,39]. Although study/treatment duration of under one year meant that discounting was not appropriate in many of the included studies, eight studies did not mention the study duration thus it could not be justified whether the discounting was applicable or not. It is encouraging that sensitivity analysis was performed by most of the studies using different parameters. Robust results were shown by six studies where mortality estimates, LoS, cost per day, and drug resistance were presented as the most sensitive parameters [27,32,34,37,38,40].

The difference in reported results between model-based and trial-based studies found here is similar to findings by Alouki and colleagues in a review of economic evidence for diabetes prevention, which suggests that there is a tendency of over-estimation in model-based studies [45]. Short study duration (often less than 30 days) and small sample size could influence the statistical significance of outcome and/or cost differences in trial-based studies, although this was not clearly the case, with several short-duration and small studies reporting significant results. Differences across studies in inclusion of cost items and valuation may also have influence on non-significant findings.

This study examined the quality of reporting in the existing economic evidence using the CHEERS checklist and found lack of compliance for many of the specified criteria within the checklist. This is similar to previous reviews of reporting quality of economic evaluation in other clinical areas [17,46].

The studies included in this review report outcome and cost data based largely on developed countries with aged people. This restricts the applicability of findings for LMIC country contexts, where children are the main vulnerable group for both morbidity and mortality due to pneumonia. The lack of information on households’ perspective on costs and outcomes suggests that incorporating a broader societal perspective and more diverse regional findings including both high and LMIC countries would support more rigorous, robust and useful comparative economic analysis.

### Strength and limitations

Key strengths of this review include the use of pre-specified systematic review methods, systematic search applied to multiple relevant databases with no restriction on publication year, and quality appraisal using the widely-accepted CHEERS tool. The review included economic evaluation studies based on a range of clinical study designs, including RCT, model-based analysis, pre-post evaluation and case report or record review studies. Despite these strengths, the study has some limitations. Firstly, difference in unit costs of the studies and assortment of outcomes preventing any quantitative pooling of results and therefore we were unable to conduct meta-analysis or present results in a single cost-effectiveness plane. Secondly, the review is limited to literature published only in English thus might not capture all relevant articles. A number of studies were excluded on the basis of language and it is possible that inclusion of these studies may have provided different economic results to those reported above. Thirdly, while multiple databases were included, searching with specific databases may cause omission of some potential articles. Finally, no attempt was made to contact the authors of the selected articles or others in the field to explore any unreported information, thereby some extent of reporting bias cannot be ignored.

## Conclusions

Comparative effectiveness with associated costs of different health care interventions for pneumonia management offers valuable insights of the cost-effectiveness of available health services that can inform policy and practice. Our review of the existing economic evidence for pneumonia management strategies demonstrates the various approaches to evaluation and study designs for economic analysis in this area. The review identified potential inventions for management of pneumonia that could be cost-effective if implemented, although mostly concentrated on comparison of different antimicrobials at a health service level rather than offering innovative management strategies to improve overall societal costs and outcomes. The current evidence is largely based on high income countries and does not address the efficiency or feasibility of interventions in resource poor settings. Reporting quality varied, with much improvement to be made in the reporting of economic analysis in the literature.

## Supporting information

S1 File Search strategy and search results for conducting systematic review of economic evaluation of management strategies of pneumonia. (DOCX)

S2 File All titles fould from sytematic search from selected databases. (XLSX)

S3 File PRISMA 2009 checklist. (DOC)
