# Supplementary material for: Economic evaluation of community acquired pneumonia management strategies: A systematic review of literature
Source: PLoS One. 2019 Oct 24;14(10):e0224170. doi: 10.1371/journal.pone.0224170 (PMC6812874; doi:10.1371/journal.pone.0224170)
Supplement: S1 File — (DOCX) [file pone.0224170.s001.docx]

## Supplementary file 1

Search results of economic evaluation of management strategies of pneumonia

| **Databases** | **#** | **Search terms** | **Subject heading/Thesaurus/limiters** | **Number of Hits** |
| --- | --- | --- | --- | --- |
| Interface-EBSCOhost Research database  *Databases:*  Academic search complete EconLIT Global Health MEDLINE Complete MEDLINE | S1 | Pneumonia | No limit | 355,248 |
|  | S2 | Pneumonia | Subject Thesaurus (pneumonia, streptococcus pneumoniae, pneumococcal vaccine, respiratory infection, pneumococcal pneumonia, antibiotics, community acquired pneumonia, hospital care, respiratory infection, respiratory disease, outcome assessment, pneumonia diagnosis) | 123,672 |
|  | S3 | Economic evaluation | No limit | 33,261 |
|  | S4 | “Economic evaluation” OR “cost effectiveness analysis” OR “cost benefit analysis” OR “cost utility analysis” OR “cost minimization analysis” OR “cost effective” OR “cost benefit” OR “cost minimization” or “cost minimisation” OR “cost comparison” | No limit | 345,616 |
|  | S5 | #S2 AND #S4 | No limit | 3,363 |
| Embase | 1 | pneumonia'/exp OR pneumonia) AND ('economic evaluation'/exp OR 'economic evaluation' OR 'cost effectiveness analysis'/exp OR 'cost effectiveness analysis' OR 'cost benefit analysis'/exp OR 'cost benefit analysis' OR 'cost utility analysis'/exp OR 'cost utility analysis' OR 'cost minimization analysis'/exp OR 'cost minimization analysis') | No limit | 2,167 |
